# BCL-2 inhibition is a promising therapeutic strategy for small cell lung cancer

**DOI:** 10.18632/oncoscience.455

**Published:** 2018-08-14

**Authors:** Timothy L Lochmann, Ynes M Bouck, Anthony C Faber

**Affiliations:** Anthony Faber: Philips Institute for Oral Health Research, Virginia Commonwealth University School of Dentistry and Massey Cancer Center, Richmond, VA, USA

**Keywords:** targeted therapy, SCLC, BCL-2 apoptosis

Extensive stage small cell lung cancer (SCLC) has a 3% 5-year survival rate, making it a devastating disease, with few treatment options. Unlike its counterpart, non-small cell lung cancer, SCLC is devoid of kinase vulnerabilities such as EGFR, ROS and ALK. Instead, SCLC survive and proliferate largely reliant on the currently undruggable MYC family of transcription factors, and through the loss of tumor suppressors p53 and Rb [1], thus underlining the need for novel therapies towards the treatment of SCLC.

The advent of BCL-2 family interacting disruptors as effective targeted therapies has provided a new arsenal of drugs in treating cancers. Blood cancers have been the clear beneficiaries so far with these drugs. Venetoclax [2], the first in-class, in-clinic and FDA-approved pure BCL-2 BH3 mimetic has dramatically improved patient care in chronic lymphocytic leukemia (CLL) [3].

In solid tumors, however, cancers appear more dependent on fellow anti-apoptotic protein BCL-xL, and to a lesser degree MCL-1, than BCL-2. Moreover, we recently demonstrated through the Genomics of Drug Sensitivity in Cancer (GDSC) high-throughput screen of ∼800 solid tumor cancer cell lines, that navitoclax, the venetoclax predecessor that also inhibits BCL-xL, had activity in a large number of SCLCs and several subgroups of solid tumors [4]. On the contrary, a follow up high-throughput screen of venetoclax in ∼800 solid tumor cancer cell lines demonstrated that only two groups of solid tumor cancers were sensitive: a subset of *MYCN*-amplified neuroblastomas [5], and a subset of high BCL-2 expressing SCLCs [1].

**Figure 1:**
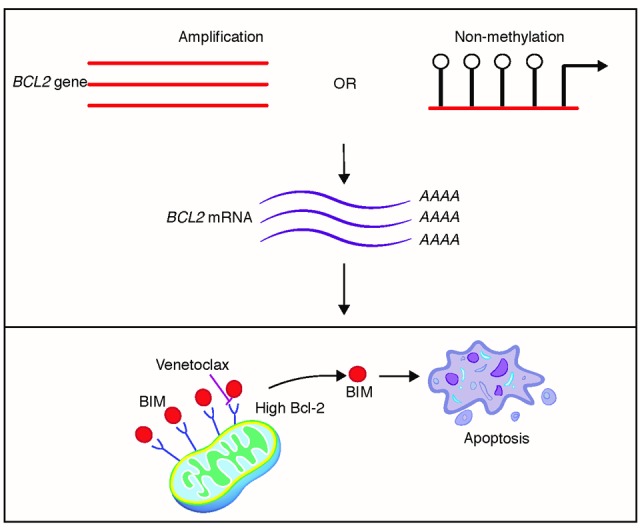
Venetoclax is effective in high BCL-2 expressing SCLC Amplification or lack of methylation of the *BCL2* gene leads to increased transcript levels of *BCL2* mRNA. Higher levels of *BCL2* mRNA correlated with increased sensitivity to the BCL-2 inhibitor, venetoclax, which displaces BIM from BCL-2, leading to apoptosis of the SCLC tumor cell.

Krystal and colleagues [6] first reported BCL-2 was overexpressed in SCLC tumors nearly 25 years ago. Since then, the hypothesis that BCL-2 contributes to SCLC survival has been studied; however, only recently with the advent of venetoclax has this hypothesis been able to be pharmacologically tested with a specific BCL-2 inhibitor. In our study, the venetoclax screen included 56 SCLC cell lines, and found that a substantial subset of SCLCs were sensitive to BCL-2 inhibition. Analyses of BCL-2 family RNA levels found that *BCL2* expression alone was best at predicting which SCLCs were the most sensitive [1]. The level of mRNA was correlated with *BCL2* amplification, or a lack of methylation in *BCL2* non-amplified SCLC cell lines (where high BCL-2 leads to greater sensitivity to venetoclax, and ultimately apoptosis) (Figure 1). While our findings of venetoclax sensitivity are in accordance with other studies that have demonstrated some SCLCs have a distinct reliance on BCL-2 [7, 8], the prospect of using BCL-2 expression alone as a surrogate marker for patients that may respond to venetoclax will likely be important for future therapeutic evaluation.

Importantly, venetoclax, orally dosed at 100 mg/kg/qd, was sufficient to induce tumor stasis or tumor regressions in several mouse models of SCLC, including chemorefractory patient-derived xenograft models. As also demonstrated in *MYCN*-amplified neuroblastomas [5], the susceptible SCLCs died via BIM- mediated apoptosis following disruption of BIM:BCL-2 complexes [1].

While these data are promising for future SCLC clinical testing, it also raises a number of questions as to how venetoclax may best be used clinically. Due to the initial chemosensitivity of SCLC tumors, venetoclax will almost certainly be introduced in the refractory population first, most likely as a combination with either topotecan or irinotecan. How venetoclax and these drugs may interact to induce unwanted hematological toxicity, and wanted tumor toxicity, remains unstudied. However, venetoclax is well tolerated even in elderly patients with severe hematological disease [3], a good indication for future combination therapies. A second question is what happens with BCL-2 dependency during chemotherapy-induced tumor progression of SCLC tumors? This has traditionally been a difficult question to answer as SCLCs have not been routinely re-biopsied.

Although these questions require further study, the demonstration that venetoclax has activity in a large portion of high BCL-2 SCLCs should provide some optimism that there may be a new therapeutic strategy on the horizon.
